# The diagnostic and prognostic value of IgG and IgA anti-citrullinated protein antibodies in patients with early rheumatoid arthritis

**DOI:** 10.3389/fimmu.2022.1096866

**Published:** 2023-01-05

**Authors:** Daniela Sieghart, Christian Konrad, Sascha Swiniarski, Helmuth Haslacher, Daniel Aletaha, Günter Steiner

**Affiliations:** ^1^ Division of Rheumatology, Department of Internal Medicine III, Medical University of Vienna, Vienna, Austria; ^2^ Thermo Fisher Scientific, Freiburg, Germany; ^3^ Department of Laboratory Medicine, Medical University of Vienna, Vienna, Austria; ^4^ Ludwig Boltzmann Institute for Arthritis and Rehabilitation, Vienna, Austria

**Keywords:** anti-citrullinated protein autoantibodies, cyclic citrullinated peptide, IgA autoantibodies, diagnostic performance, rheumatoid arthritis

## Abstract

**Objectives:**

Anti-citrullinated peptide antibodies (ACPA) are specific markers for rheumatoid arthritis (RA) and typically measured by assays employing a cyclic citrullinated peptide (CCP) as antigen. This study was aimed at investigating the diagnostic performance of anti-CCP2 and anti-CCP3 IgG and IgA assays in patients with early RA with a particular focus on the potential prognostic value of IgA ACPA.

**Methods:**

The anti-CCP3.1 assay (Inova Diagnostics) measuring IgG and IgA antibodies simultaneously was compared to anti-CCP2 IgG and IgA assays (Thermo Fisher Scientific) employing sera of 184 early RA patients, 360 disease controls and 98 healthy subjects.

**Results:**

Anti-CCP2 IgG and IgA assays showed high specificity versus disease controls (98.9%; 99.4%). Sensitivity was 52.2% (IgG) and 28.8% (IgA), resulting in positive likelihood ratios (LR+) of 47.5 (IgG) and 48.0 (IgA). The anti-CCP3.1 assay proved slightly more sensitive than the anti-CCP2 IgG assay (56%) but specificity was markedly lower (90.8% versus disease controls). However, when using a threefold higher cut-off specificity of the anti-CCP3.1 assay increased (97.5%) while sensitivity (52.7%) became comparable to the anti-CCP2 IgG assay resulting in a LR+ of 21.5. Anti-CCP2 IgA antibodies did not increase the diagnostic sensitivity of ACPA testing, but IgA positive patients showed diminished responses to treatment with anti-TNF biologicals compared to patients who had only IgG antibodies.

**Conclusion:**

Specificity of ACPA assays should be adjusted to reduce the risk of misclassification and a false positive diagnosis. Determination of ACPA IgA might provide important prognostic information concerning therapeutic responses.

## Highlights

- Anti-CCP2 and anti-CCP3 assays show equivalent diagnostic performance in early RA patients when adjusted for high specificity. Determination of IgA anti-citrullinated protein antibodies might provide additional prognostic information with respect to therapeutic responses.

## Introduction

Besides rheumatoid factor (RF), anti-citrullinated protein antibodies (ACPA) are the most important serological markers for rheumatoid arthritis (RA). ACPA are predominantly of the immunoglobulin (Ig)G isotype and are more specific than IgM-RF. They are commonly determined by assays using a cyclic citrullinated peptide (CCP) as antigen. Since their first description (1), different generations of CCP based assays (CCP1-CCP3) have been developed which show some differences regarding sensitivities and specificities (2–4). Serological testing for ACPA and RF is particularly important in early disease stages and has been incorporated in the 2010 ACR/EULAR classification criteria for RA (5). This may have considerable impact on clinical decision making because patients with high titer antibodies would be more readily diagnosed as RA (6, 7). However, ACPA show only moderate sensitivity in early RA which is comparable to sensitivity of RF. Thus, about one third of early RA patients are seronegative for ACPA and/or RF. Therefore reducing the serological gap is an important issue in RA sero-diagnostics and several studies have tried to achieve this by determining additionally IgA isotypes of RF and ACPA (8–11). However, the overall sensitivity of ACPA testing could not be substantially increased and therefore routine diagnostics still rely on anti-CCP IgG assays.

Nevertheless, an assay has been developed (anti-CCP3.1) that detects IgG and IgA ACPA isotypes simultaneously and may have superior sensitivity compared to anti-CCP2 assays that measure only the IgG isotype (12, 13). However, it is unclear if this increased sensitivity is based on inclusion of IgA antibodies or rather on the nature of the CCP3 peptides which might be more sensitive for ACPA detection than the peptides contained in the CCP2 assay (14, 15).

Therefore this study aimed to investigate the diagnostic performance of the anti-CCP3.1 assay in comparison to anti-CCP2 IgG and IgA assays in patients with early RA and an appropriate number of disease controls. Moreover, we addressed the potential prognostic value of ACPA IgA determination, especially with respect to therapeutic responses to disease modifying anti-rheumatic drugs.

## Methods

### Patients

Sera were routinely obtained from patients with early RA, classified according to the 2010 ACR/EULAR criteria (3), recruited during their regular visits at the outpatients clinic at the Division of Rheumatology of the Medical University of Vienna. Patients presented with a median symptom duration of 0.2 years. The demographic data of the 184 patients selected retrospectively for this study are shown in **Supplementary Table 1**. All clinical information was stored in a database including >4.000 patients with RA. Treatment information was available for all patients and used to calculate drug survival times to methotrexate (MTX) (n=144) and the first anti-TNF biological therapy (n=142): 78 patients were treated with Adalimumab, 38 with Etanercept, 14 with Golimumab and 12 patients received Infliximab; out of these 142 patients 22 received an anti-TNF monotherapy. Treatment responses after 3 months were calculated using the simplified disease activity index (SDAI)50, referring to a 50% improvement in SDAI. In addition, x-rays from hands and feet of 140 RA patients were analysed for radiographic progression using the Sharp/van der Heijde (SvdH) score. A mean annual progression rate was calculated out of multiple timepoints and was compared within groups; 32% of patients showed an erosive disease course with >5 mean annual progression rate.

Sera from 360 patients with other rheumatic diseases were collected from 95 patients with osteoarthritis (OA), 92 patients with systemic lupus erythematosus (SLE), 48 patients with ankylosing spondylitis (SpA), 45 patients with systemic sclerosis, 13 patients with reactive arthritis, 19 patients with primary Sjögren´s syndrome, 15 patients with autoimmune inflammatory myopathies, 14 patients with granulomatosis with polyangiitis and 19 patients with a diagnosis of osteoporosis. Sera from 98 healthy subjects were collected during voluntary health examination offered by the Austrian social insurance. Disease controls had a median age of 55 (43–64) years and 68.8% were females. Healthy controls had a median age of 50 (42.5–55) years and 72% were females. The study was approved by the ethics committee of the Medical University of Vienna (ethics vote numbers: 559/2005 and 2002/2014). Biomaterial was processed and stored until analysis according to standard operating procedures by the Medical University Vienna biobank, a central facility included in a certified quality management system (16).

### Antibody detection

Sera were analysed for the presence of anti-CCP antibodies by anti-CCP2 IgG and IgA assays (EliA™ CCP, Thermo Fisher Scientific) and the combined IgG/IgA anti-CCP3.1 assay (Quanta Lite^R^CCP3.1, Inova Diagnostics). Cut-offs recommended by the manufacturers were 10 arbitrary units (AU)/ml for anti-CCP2 IgG and IgA and 20 AU/ml for anti-CCP3.1. Regarding the anti-CCP3.1 assay, a cut-off of 60 AU/ml (high positive according to the manufacturer) was additionally employed.

### Statistical analysis

Data are shown as median and interquartile ranges (IQR). Nonparametric statistical methods were used for comparisons. Kruskal-Wallis test for comparing continuous variables between groups and Fisher’s exact test for differences in dichotomous variables. Specificities as well as positive likelihood ratios (LR+) were calculated either against healthy controls or disease controls. MTX and anti-TNF retention rates were calculated and presented using Kaplan-Meier curves.

All statistical analysis was performed using SPSS (version 28). A P value of less than 0.05 was considered to indicate statistical significance.

## Results

### Sensitivity and specificity

As expected, anti-CCP2 IgG and IgA assays showed very high specificities ≥ 98% both versus healthy subjects and disease controls. Sensitivities were 52.2% for IgG and 28.8% for IgA antibodies, respectively. This resulted in high positive likelihood ratios (LR+) versus disease controls of 47.5 for the IgG and 48.0 for the IgA assay (**Table 1**). However, IgA antibodies did not show an added diagnostic value in our early RA cohort as all IgA positive patients were also IgG positive. The anti-CCP3.1 assay was found to be slightly more sensitive than the anti-CCP2 IgG assay with 56% of early RA patients testing positive. However, specificity was lower amounting to 95.9% versus healthy subjects and 90.8% versus disease controls which resulted in a relatively low LR+ of 13.7 vs. healthy subjects and 6.1 vs. disease controls, respectively (**Table 1**).

**Table 1:** Diagnostic performance of the anti-CCP2 IgG, CCP2 IgA and the CCP3.1 IgG/IgA assays.

	CCP2 IgG	CCP2 IgA	CCP3.1	CCP3.1
cut-off (AU/ml)	10	10	20	60
patients positive	96	53	103	97
Sensitivity	52.2%	28.8%	56.0%	52.7%
number of pos healthy controls	1	2	4	1
specificity (healthy)	99.0%	98.0%	95.9%	99.0%
LR+ (healthy)	52.2	14.4	13.7	52.7
number of pos disease controls	4	2	33	9
specificity (disease controls)	98.9%	99.4%	90.8%	97.5%
LR+ (disease controls)	47.5	48.0%	6.1	21.1

Sera from 184 RA patients, 360 disease controls and 98 healthy subjects were analysed to calculate sensitivity, specificity and LR+ of the three assays. Cut-offs were employed according to the manufacturer’s instructions (AU = arbitrary units). Regarding the anti-CCP3.1 assay the cut-off defined by the manufacturer as high positive (60 AU/ml) was used to recalculate assay performance.

Thus, out of 360 disease controls 33 patients (9.2%) were found to be positive for anti-CCP3.1 whereas only 4 control patients (1.1%) were anti-CCP2 IgG positive; of these 2 patients (0.6%) had also IgA antibodies (**Table 2**). Among anti-CCP3.1 positive disease controls the most common diagnoses were osteoarthritis (n=12), ankylosing spondylitis (n=6) and SLE (n=5); 6 patients suffered from other autoimmune rheumatic diseases, two patients were diagnosed with reactive arthritis and two had osteoporosis. Furthermore, 4 healthy controls were positive in the CCP3.1 assay of which only one was also positive in the CCP2 assays (**Table 2**).

**Table 2:** Non-RA patients and healthy subjects showing a positive result in anti-CCP3.1 IgG/IgA and/or anti-CCP2 IgG and IgA testing.

Diagnosis	CCP3.1 IgG/IgA	evaluation	CCP2 IgG	evaluation	CCP2 IgA	evaluation
Osteoporosis	22	**+**	1.0	–	0.6	–
Healthy subject	22	**+**	1.1	–	1.5	–
Ankylosing spondylitis	24	**+**	1.4	–	1.8	–
Osteoarthritis	25	**+**	0.7	–	1.1	–
SLE	26	**+**	18.8	**+**	2.3	–
SLE	27	**+**	1.6	–	1.8	–
Osteoarthritis	28	**+**	0.6	–	1.7	–
Ankylosing spondylitis	28	**+**	1.0	–	1.3	–
Osteoarthritis	29	**+**	1.5	–	8.5	–
Ankylosing spondylitis	30	**+**	0.8	–	1.1	–
SLE	31	**+**	1.9	–	2.0	–
Systemic sclerosis	31	**+**	2.1	–	2.6	–
Myositis	34	**+**	3.1	–	0.0	–
Osteoporosis	37	**+**	1.5	–	3.3	–
Healthy subject	37	**+**	1.0	–	0.7	–
Osteoarthritis	38	**+**	6.3	–	1.4	–
Reactive arthritis	38	**+**	1.0	–	1.8	–
Osteoarthritis	38	**+**	1.1	–	1.1	–
Osteoarthritis	39	**+**	1.0	–	1.1	–
Osteoarthritis	40	**+**	1.0	–	2.0	–
Ankylosing spondylitis	46	**+**	1.5	–	1.4	–
Healthy subject	47	**+**	0.7	–	0.3	–
Osteoarthritis	51	**+**	0.8	–	1.8	–
Granulomatosis with polyangiitis	52	**+**	0.9	–	0.8	–
Osteoarthritis	52	**+**	0.9	–	0.8	–
Ankylosing spondylitis	54	**+**	0.7	–	1.2	–
Systemic sclerosis	55	**+**	1.1	–	2.0	–
Osteoarthritis	86	**+**	1.8	-	0.8	-
Systemic sclerosis	122	**+**	1.1	-	1.7	-
Osteoarthritis	137	**+**	1.3	-	1.8	-
Osteoarthritis	157	**+**	1.1	-	1.0	-
SLE	160	**+**	1.7	-	1.7	-
Sjögren´s syndrome	174	**+**	1.1	-	0.8	-
Reactive arthritis	250	**+**	585.3	**+**	52.8	**+**
Ankylosing spondylitis	250	**+**	166.2	**+**	11.1	**+**
SLE	250	**+**	99.4	**+**	4.3	-
Healthy subject	250	+	203.3	+	62.1	+

Rows marked in grey identify those disease controls which would still be positive in the anti-CCP3.1 assay when the high positive (60 AU/ml) cut-off is applied.

**Figure 1:**
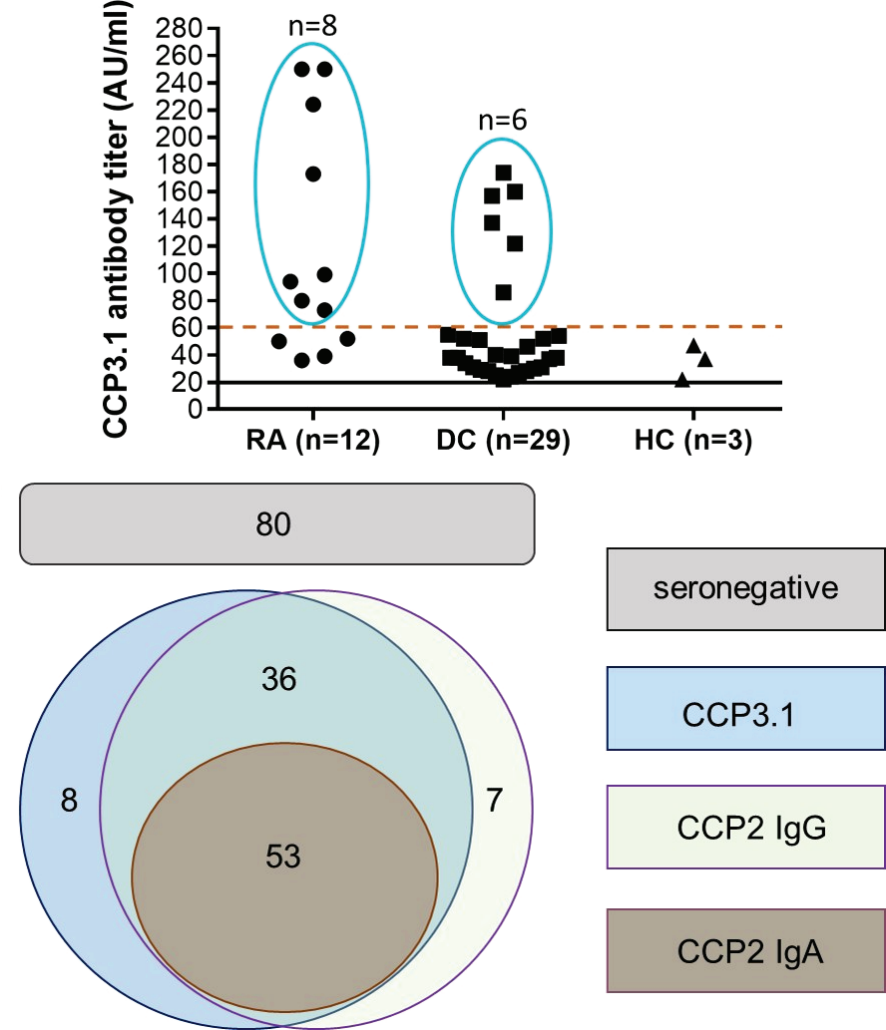
Anti-CCP2 IgG, CCP2 IgA and CCP3.1 IgG/IgA antibodies in early RA patients and disease controls. **(A)** Anti-CCP3.1 antibody levels in anti-CCP3.1 positive/anti-CCP2 IgG negative early RA patients, disease controls (DC) and healthy controls (HC). When applying a cut-off level of 60 AU/ml (high positive), the number of positive disease controls and healthy controls was reduced from 29 to 6 and from 3 to 0, respectively, while 8 out of the 12 anti-CCP2 negative early RA patients remained positive. **(B)** Schematic representation of the overlap of anti-CCP2 IgG, anti-CCP2 IgA and anti-CCP3.1 IgG/IgA positivity in the early RA cohort when applying the high positive cut-off (60 AU/ml) for the anti-CCP3.1 assay. Out of 184 patients, 53 were found being triple positive for anti-CCP2 IgG, IgA and anti-CCP3.1, 36 patients were double positive for anti-CCP2 IgG and anti-CCP3.1; 7 patients were solely positive for anti-CCP2 IgG and 8 patients for anti-CCP3.1. Eighty patients were negative in all assays (seronegative).

Since the majority of false positive patients had low level anti-CCP3.1 antibodies we re-analysed the data using a cut-off value of 60 AU/ml (high positive as defined by the manufacturer, see Methods). This resulted in an increased specificity of 99% versus healthy subjects and of 97.5% versus disease controls with a LR+ of 52.7 versus healthy subjects and 21.1 versus disease controls. Only 9 out of 33 control patients remained positive (3 osteoarthritis, 1 ankylosing spondylitis, 1 reactive arthritis and 4 with other autoimmune inflammatory diseases). In contrast, two thirds out of the 12 anti-CCP3.1 positive early RA patients testing negative in the anti-CCP2 assay showed levels above 60 AU/ml (**Figure 1A**). Thus, at this elevated cut-off sensitivity was only slightly reduced (and virtually identical to the anti-CCP2 IgG assay) while the LR+ increased 3.5-fold versus disease controls and 3.8-fold versus healthy controls.

Taken together, when applying the high cut-off, 53 early RA patients were triple positive for anti-CCP2 IgG, anti-CCP2 IgA and anti-CCP3.1; 36 patients were double positive for anti-CCP2 IgG and anti-CCP3.1; 7 patients were solely positive for anti-CCP2 IgG and 8 patients solely for anti-CCP3.1. Overall sensitivity of the two IgG assays was 56.5% with 80 patients remaining seronegative for anti-CCP antibodies (**Figure 1B**).

### Prognostic value of ACPA IgA

**Figure 2:**
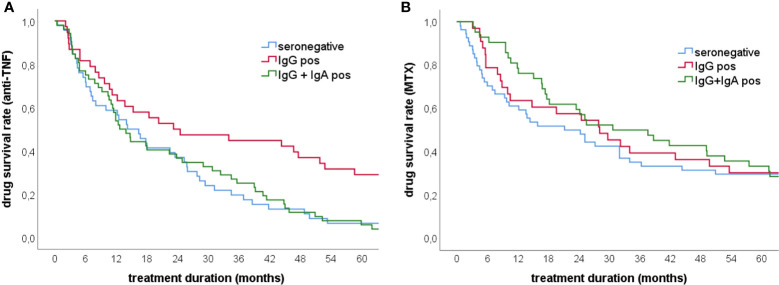
Drug survival rates for anti-TNF and methotrexate treatment in early RA patients. **(A)** Anti-TNF survival rates in patients showing anti-CCP2 IgG/IgA antibodies (n=53), anti-CCP2 IgG antibodies (n=43) and seronegative patients (n=46) **(B)** Methotrexate survival rates in patients showing anti-CCP2 IgG/IgA antibodies (n=47), anti-CCP2 IgG antibodies (n=43) and seronegative patients (n=54). The presence of IgA antibodies was significantly associated with reduced anti-TNF retention rates while for MTX retention no significant difference was found between the groups.

Although anti-CCP2 IgA antibodies did not increase the diagnostic specificity of ACPA testing, they might have some prognostic value as has been proposed for RF IgA (17). To address this issue we calculated drug retention rates for MTX and anti-TNF biologicals. These analyses revealed decreased drug survival times for anti-TNF biologicals in patients with anti-CCP2 IgA antibodies (**Figure 2A**). The difference between anti-CCP2 IgG single positive and IgG/IgA double positive patients was statistically significant at 18 months and persisted until the end of the observation period (60 months). After one year, 65% of anti-CCP2 IgG single positive patients were on anti-TNF treatment as compared to 50% of patients with anti-CCP2 IgG/IgA antibodies. After three years 44% of the IgG single positive patients but only 23% of IgG/IgA double positive patients were still under anti-TNF treatment. A reduced retention rate was also observed in seronegative patients of which only 19% remained on anti-TNF therapy. Five years after treatment initiation still approximately 30% of anti-CCP2 IgG single positive patients were taking anti-TNF biologicals whereas almost 95% of patients with anti-CCP2 IgG/IgA antibodies had discontinued their first anti-TNF biological, similar to seronegative patients. As expected, such prognostic value could not be attributed to the CCP3.1 assay which measures IgG and IgA antibodies simultaneously (**Supplementary Figure 1**).

Regarding the response to anti-TNF treatment after 3 months there was no significant difference in SDAI50 between patients with anti-CCP2 IgG and those with IgG/IgA antibodies. In addition, there was also no significant difference between IgG single positive patients and IgG/IgA double positive patients regarding disease activity at baseline (**Supplementary Table 1**) as well as during the disease course.

Concerning baseline treatment with MTX, no statistically significant differences between the three patient groups were observed although during the first year MTX survival rate appeared to be higher in patients with anti-CCP2 IgA antibodies (**Figure 2B**).

Mean radiographic progression rate was higher in anti-CCP positive patients compared to seronegative patients. Interestingly however, the difference in progression rates reached the level of statistical significance only for IgG/IgA positive patients although no significant difference was seen between anti-CCP2 IgG single positive and anti-CCP2 IgG/IgA double positive patients (**Supplementary Figure 2**).

## Discussion

ACPA are undoubtedly the most specific serological markers of RA and according to the classification criteria would be strongly indicative of RA, particularly in case of high titer (5). However, the prevalence of ACPA in early RA does usually not exceed 55% and therefore manufacturers of diagnostic assays have attempted to increase the sensitivity of ACPA assays. Since most commercial assays are employing the CCP2 peptides as antigen (and therefore show similar performance) efforts have been undertaken to increase sensitivity of ACPA testing by using other antigens such as mutated citrullinated vimentin (18, 19), citrullinated viral peptides (20) or another artificial peptide mixture such as CCP3 (13–15). Moreover, additional determination of ACPA IgA isotypes has been suggested but proven to only marginally increase overall sensitivity of ACPA testing (8–10).

In line with some previous reports (6, 21) we confirmed that in our cohort of early RA patients the anti-CCP3.1 assay showed indeed a slightly higher sensitivity than the anti-CCP2 IgG assay. However, this was not due to the simultaneous measurement of IgG and IgA isotypes but rather due to the low cut-off of the assay. Thus, the moderate gain in sensitivity was obtained at the expense of specificity which was significantly reduced both versus disease controls and healthy subjects confirming observations made also by other researchers (3, 6). This issue has been addressed in some detail in a recent study by van Hoovels et al. in which commercial assays for ACPA and RF were compared, particularly in respect of their impact on RA classification (7).

When employing a three-fold higher cut-off (as suggested by the manufacturer), diagnostic performance of the anti-CCP3.1 assay became comparable to the anti-CCP2 assay since 24 disease controls (but only 6 early RA patients) became negative. Therefore, anti-CCP3.1 levels below 60 AU/ml did not appear to be meaningful or could even be misleading because the majority of patients with low antibody levels (33 out of 38) did not have a diagnosis of RA (**Table 2**). This was obviously also the case for the four anti-CCP3.1 positive healthy controls among which only one had high titer antibodies. Remarkably, the serum showed also high levels of anti-CCP2 IgG and IgA antibodies and therefore this healthy subject might be at risk for developing RA (6).

Although at the higher cut-off the agreement between the two assays was very good, 8 patients were solely positive for anti-CCP3.1 while 7 patients were solely positive for anti-CCP2 (**Figure 1B**). This may reflect the different nature of the two peptides and/or the technical differences between both test methods (4) as the anti-CCP2 IgG assay is an automated and random access fluoroenzyme immunoassay (FEIA) compared to the manual enzyme-linked immunosorbent assay (ELISA) used to detect anti-CCP3.1 antibodies.

Clearly, no single assay will detect all ACPA positive patients but users should be aware of specificity and adjust cut-off values accordingly to obtain at least 98% specificity vs healthy controls. Our data also confirm that measuring IgA antibodies does not increase the diagnostic sensitivity of anti-CCP testing. However, ACPA IgA may further increase the diagnostic specificity because in accordance with previous investigations only two disease controls and one healthy subject showed this type of dual IgG/IgA reactivity (10). Interestingly, in a recent study a broad autoantibody profile at baseline including also ACPA IgA and IgM isotypes was associated with an increased early response to treatment with MTX but not with long-term outcome (22). This appeared also to be the case in our cohort of early RA patients although statistical significance was not obtained (**Figure 2B**). In contrast, with respect to anti-TNF biologicals patients with anti-CCP2 IgG/IgA antibodies showed a significantly reduced anti-TNF retention rate as compared to patients who had only IgG antibodies or seronegative patients. Interestingly, in seronegative patients a reduced retention rate has been reported for Abatacept suggesting that the presence of ACPA and/or RF could be used as an indicator for a more long-lasting response to biological drugs (23). Thus, it will be interesting to see if Abatacept retention rates are also affected by IgA antibodies, a matter that would deserve further investigations. However, it should be borne in mind that an association of ACPA (IgG) or RF (IgM) with the clinical treatment response to TNF inhibitors has not been observed (24) and also in our cohort no significant difference was seen between the three patient groups with respect to anti-TNF treatment responses as measured by SDAI.

The prognostic value of IgA anti-CCP2 appeared to be long lasting because 5 years after treatment initiation, only 5% of IgG/IgA positive patients were still under anti-TNF treatment as compared to 30% of IgG positive patients. This might have some impact on clinical decision making and favour treatment of ACPA and/or RF IgA positive patients with a B- or T cell targeting therapy. Our finding is also in line with a previous study in which the presence of RF IgA was associated with a blunted response to treatment with TNF inhibitors (17). Of note, the vast majority of our anti-CCP2 IgG/IgA positive early RA patients was also positive for RF IgA (**Supplementary Table 1**). Furthermore, in a recently published study ACPA IgA was identified as a risk factor for disease flare during drug tapering in patients with RA (25) and there is now emerging eveidence that IgA autoantibodies, and particularly the IgA2 subclass, contribute to the inflammatory processes of RA by activating macrophages and neutrophils (26). Taken together, these data further support the arguments for testing separately for RF and ACPA IgA isotypes because these assays can provide valuable information which could allow further stratification of RA patients.

In summary, the two ACPA assays under investigation showed comparable performance in patients with early RA when the cut-off of the CCP3.1 assay was adjusted to ensure high specificity. Of note, in our early RA cohort overall sensitivity of the CCP2 IgG and CCP3.1 assay amounted to 56.5% as compared to approximately 52% of the individual assays. Therefore, use of both assays might be considered to increase overall sensitivity of ACPA testing without affecting specificity. Moreover, the use of both assays could also improve diagnostic accuracy, particularly in patients at risk for developing RA (6, 13, 27). Although this would increase the costs of ACPA testing the gain of identifying additional RA patients in the earliest stages of disease would justify such strategy because the costs of a delayed diagnosis and treatment in a false negative patient would by far outmatch the relatively low costs of one additional assay that needs to be done only at baseline (28).

Since approximately 10% of anti-CCP2/CCP3.1 negative patients tested positive for RF IgM (and most of them also for RF IgA) overall sensitivity of ACPA and RF testing in our cohort amounted to approximately 67%. While compared to ACPA specificity of RF IgM is moderate, especially in the absence of ACPA, combined presence of RF IgM/IgA has been found to be more specific for RA arguing again for including also RF IgA into routine diagnostics (10, 11). Interestingly, the seven CCP2 IgG positive/CCP3.1 negative sera were all positive for RF IgM as compared to only four of the eight CCP3.1 positive/CCP2 negative sera. Even though this may be pure coincidence such observation would deserve further investigation in larger cohorts of RA patients.

For the future, assays containing a mixture of citrullinated, carbamylated and acetylated peptides might indeed reduce the serological gap (29) and further increase specificity of ACPA testing because the presence of multiple reactivities has been reported to be highly specific for RA (10, 30). Furthermore, determination of IgA isotypes should be taken into serious consideration because their presence might have considerable impact on therapeutic decision making which needs to be addressed in more elaborate multi-centre studies.

## Data availability statement

The original contributions presented in the study are included in the article/**Supplementary Material**. Further inquiries can be directed to the corresponding author.

## Ethics statement

The studies involving human participants were reviewed and approved by Ethics committee of the Medical University of Vienna. The patients/participants provided their written informed consent to participate in this study.

## Author contributions

GS: Study design, data interpretation, manuscript writing. DS: Study design, data acquisition, data interpretation, preparation of figures, manuscript writing. CK and SS: Study design, data acquisition, data interpretation. HH: Data acquisition. DA: Data interpretation, manuscript writing. All authors contributed to the article and approved the submitted version.
