# Supplementary material for: The diagnostic and prognostic value of IgG and IgA anti-citrullinated protein antibodies in patients with early rheumatoid arthritis
Source: Front Immunol. 2023 Jan 5;13:1096866. doi: 10.3389/fimmu.2022.1096866 (PMC9849943; doi:10.3389/fimmu.2022.1096866)
Supplement: Supplementary file 1 [file DataSheet_1.docx]

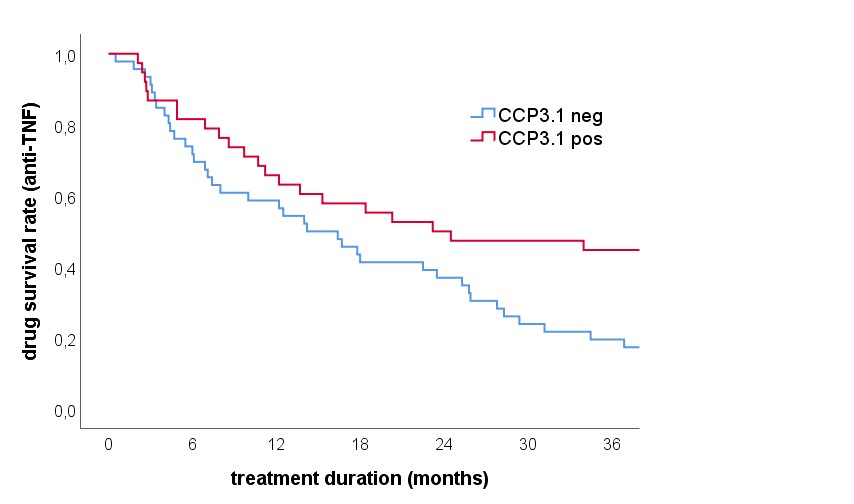


**Supplementary Figure 1.** Drug survival rates for anti-TNF treatment in anti-CCP3.1 positive and anti-CCP3.1 negative early RA patients
