# Supplementary material for: The diagnostic and prognostic value of IgG and IgA anti-citrullinated protein antibodies in patients with early rheumatoid arthritis
Source: Front Immunol. 2023 Jan 5;13:1096866. doi: 10.3389/fimmu.2022.1096866 (PMC9849943; doi:10.3389/fimmu.2022.1096866)
Supplement: Supplementary file 2 [file DataSheet_2.docx]

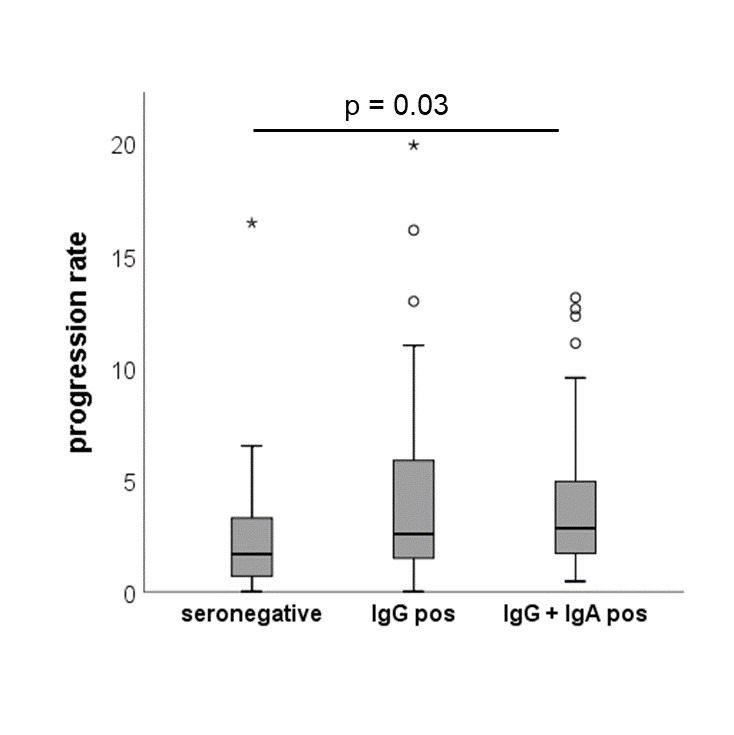


**Supplementary Figure 2.** Mean annual progression rate of anti-CCP2 IgG single positive, anti-CCP2 IgG/IgA double positive and anti-CCP2 negative early RA patients.

A siginifcant difference was found between double positive and seronegative patients. There was neither a significant difference between IgG single positive patients to seronegative patients nor between the single positive and double positive patients. Outliers are indicated by an asterisk*.
