# Supplementary material for: The diagnostic and prognostic value of IgG and IgA anti-citrullinated protein antibodies in patients with early rheumatoid arthritis
Source: Front Immunol. 2023 Jan 5;13:1096866. doi: 10.3389/fimmu.2022.1096866 (PMC9849943; doi:10.3389/fimmu.2022.1096866)
Supplement: Supplementary file 3 [file Table_1.docx]

**Supplementary Table S1.** Baseline characteristics of early RA patients according to their anti-CCP antibody status.

|  | **Anti-CCP2 IgG positive** n=43 | **Anti-CCP2 IgG/IgA positive**  n=53 | **Anti-CCP* Negative** n=76 |
| --- | --- | --- | --- |
| age (years) | 58 (45-66) | 56 (47-64) | 56 (46-64) |
| female % | 80 | 60 | 79 |
| disease duration (years) | 0 (0-0.5) | 0 (0-0.1) | 0.2 (0-0.8) |
| clinical disease activity index (CDAI) | 12.6 (9.1-18.6) | 14.1 (7.8-21.8) | 15.1 (8.5-19.89 |
| disease activity score 28 (CRP) | 3.7 (3.2-4.4) | 4.2 (2.9-4.9) | 4.3 (3.0-4.7) |
| radiographic score (SvdH) | 5 (1.5-17) | 5 (3.7-8.2) | 8 (5-12) |
| C-reactive protein [mg/dl] | 0.6 (0.3-1.7) | 1.2 (0.5-4) | 1.1 (0.2-7.0) |
| pain (visual analog scale) | 44 (27-55) | 43 (17-62) | 40 (25-56.5) |
| patient global disease activity | 46 (27-61) | 44 (20-61) | 45 (23-53.5) |
| evaluator´s global disease activity | 16 (10-34) | 23 (9-41) | 20 (11-32) |
| health access and quality index | 0.3 (0-1.1) | 0.8 (0.1-1.2) | 0.6 (0.1-1.3) |
| swollen joint count (SJC) 28 | 3 (2.0-6.0) | 4 (1.0-8.0) | 3 (2.0-4.0) |
| tender joint count (TJC) 28 | 4 (1.0-8.0) | 2 (1.0-5.0) | 4 (1.0-6.5) |
| rheumatoid factor IgM pos % | 93.0% | 96.2% | 10.5% |
| rheumatoid factor IgA pos % | 65.1% | 90.6% | 7.9% |

* Negative for anti-CCP2 and anti-CCP3.1 antibodies (low cut-off)
